# Complexes of the Mycotoxins Citrinin and Ochratoxin A with Aluminum Ions and their Spectroscopic Properties

**DOI:** 10.3390/toxins10120538

**Published:** 2018-12-14

**Authors:** Julia Keller, Daniel Moldenhauer, Liam Byrne, Hajo Haase, Ute Resch-Genger, Matthias Koch

**Affiliations:** Division of Organic Trace and Food Analysis, Department of Analytical Chemistry, Reference Materials, Bundesanstalt für Materialforschung und-prüfung (BAM), Richard-Willstätter-Str. 11, D-12489 Berlin, Germany; juliakeller19@yahoo.de (J.K.); daniel.moldenhauer@bam.de (D.M.); c14506717@mydit.ie (L.B.); haase@tu-berlin.de (H.H.); ute.resch@bam.de (U.R.-G.)

**Keywords:** complexation, aluminum, fluorescence, Job plot, HPLC-DAD/FLD

## Abstract

The sensitive detection of the mycotoxin citrinin (CIT) utilizing its fluorescence requires approaches to enhance the emission. In this respect, we studied the complexation of CIT and ochratoxin A (OTA) with Al^3+^ in methanol using absorption and fluorescence spectroscopy. In this context, an isocratic high performance liquid chromatography (HPLC) method using a polymer column and a fluorescence detector was also developed that enables the separation of the metal ion complexes from the free ligands and non-complexed Al^3+^. CIT and OTA showed distinct changes in their absorption and fluorescence properties upon Al^3+^-coordination, and the fluorescence of CIT was considerably enhanced. Analysis of the photometrically assessed titration of CIT and OTA with Al^3+^ using the Job plot method revealed 1:2 and 1:1 stoichiometries for the Al^3+^ complexes of CIT (Al:CIT) and OTA (Al:OTA), respectively. In the case of CIT, only one β-diketone moiety participates in Al^3+^ coordination. These findings can be elegantly exploited for signal amplification and provide the base to reduce the limit of detection for CIT quantification by about an order of magnitude, as revealed by HPLC measurements using a fluorescence detector.

## 1. Introduction

The often co-occurring and nephrotoxic mycotoxins citrinin (CIT) and ochratoxin A (OTA) are produced by different fungi of the genera *Aspergillus*, *Monascus* and *Penicillium* [1,2,3]. CIT and OTA are frequently found in cereals, animal and plant products, food, and feed. They present a threat for human and animal health because of their chronic toxic effects [4,5]. For OTA, maximum levels are set by international legislation, whereas the CIT levels have not been regulated yet. For the quantification of the inherently fluorescent mycotoxins OTA and CIT in real samples, often high performance liquid chromatography with fluorescence detection (HPLC-FLD) is employed [6,7,8,9]. In addition, analytical techniques like HPLC-tandem mass spectrometry (HPLC-MS/MS) and enzyme-linked immunosorbent assays (ELISA) are commonly used for OTA and CIT determinations [10,11].

The absorption and fluorescence properties of almost every optically active species are responsive to the species’ environment, including polarity, viscosity, pH, and the presence of additives like detergents or certain coordinating metal ions [12]. This can lead to strong spectral shifts and changes particularly in fluorescence intensity/quantum yield and lifetime. This sensitivity of fluorescence can be deleterious for the spectroscopic determination and quantification of an emissive analyte, but it can be also utilized for developing signal enhancement strategies. Common examples for exploiting such a fluorescence enhancement in environment and food analysis are post-column derivatization methods for HPLC that utilize a cation coordination-induced increase in fluorescence quantum yield [13,14,15]. Metal ions frequently used for this purpose are light cations like magnesium or aluminum, thereby preventing a possible fluorescence quenching by heavy atom effects [12]. This was exploited, for example, for the mycoestrogen zearalenone in a HPLC method with post-column derivatization involving Al^3+^ chelation of this mycotoxin [13]. It is also known that the fluorescence of OTA can be enhanced by adding magnesium ions [16]. Nakazato et al. demonstrated that the fluorescence of CIT increases after adding aluminum chloride and applied this finding to the fluorometric determination of CIT in cereals [17]. In another study, it was shown using X-ray analysis that OTA forms stable complexes with several alkaline earth ions [18]. Complexing agents used for enhancing fluorescence signals in thin-layer and liquid chromatography are aluminum nitrate and chloride. Al^3+^ is an octahedrally hexacoordinated ion that binds to one, two, or three bidentate ligands, leading to the formation of 1:1, 1:2, or 1:3 complexes. It was demonstrated for studies in ethanol that small amounts of water can interfere with the complex formation. Hence, absolute methanol or ethanol are the most suitable solvents to investigate such complexation reactions [19,20].

The aim of the present study was to assess the chemical composition and stoichiometry of the barely investigated metal ion complexes of the two mycotoxins CIT and OTA and to obtain new insights into their spectroscopic properties and achievable fluorescence enhancement factors. Here, we show a detailed spectroscopic investigation of the Al^3+^ complexes of CIT and OTA, involving the measurement of absorption and fluorescence spectra, fluorescence quantum yields, and fluorescence lifetimes. The stoichiometry of the complexes was derived from the absorption spectra obtained at varying mycotoxin-to-Al^3+^ concentration ratios using the Job plot method [21]. As a prerequisite for this study, a HPLC-FLD method to separate complexes from free mycotoxin and aluminum was developed.

## 2. Results and Discussion

### 2.1. Coordination-Induced Changes in Absorption and Complex Stoichiometry

**Figure 1:**
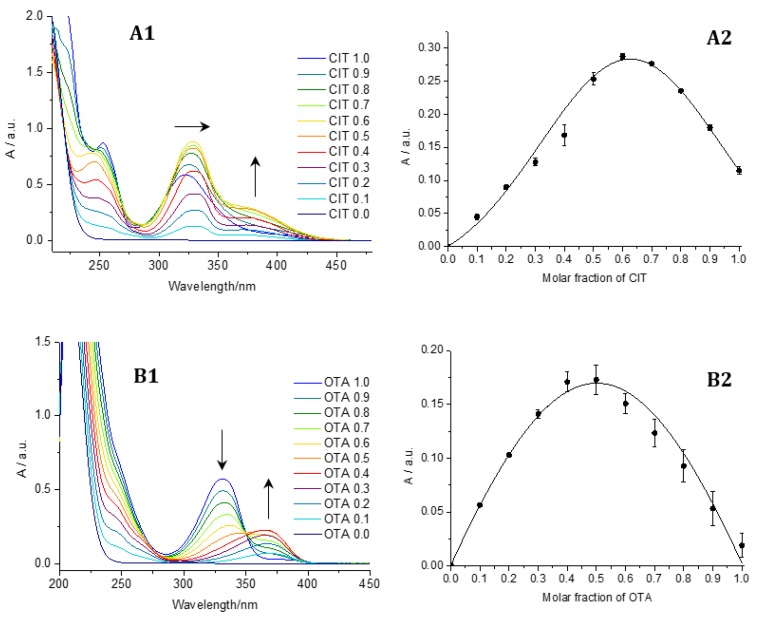
UV/VIS absorption spectra accompanying the complexation of citrinin (**A1**) and ochratoxin A (**B1**) with Al^3+^ in methanol obtained with the Job plot method. Numbers in the spectra represent the molar fractions of citrinin and ochratoxin A after addition of Al^3+^ with a concentration of 100 µM per sample (detailed information given in Section 4.5). The Job plot obtained at λmax = 365 nm (**A2**,**B2**). Black dots: observed absorbances and error bars representing the relative standard deviation; black line: Gaussian fit of the data points. CIT: citrinin; OTA: ochratoxin A.

**Figure 2:**
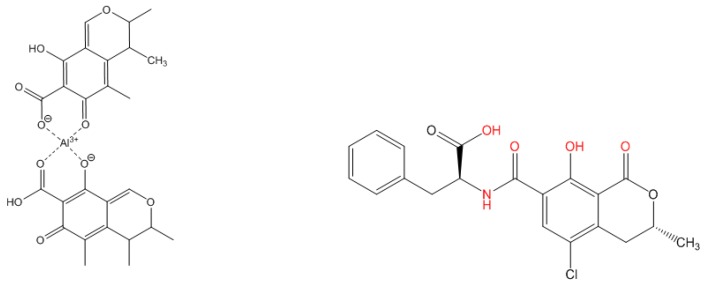
Structures of the citrinin-Al complex (left) and the ochratoxin A (right) with possible chelation sites shown in red.

Adding Al^3+^ to CIT and OTA in methanol led to a strong bathochromic shift in absorption (Figure 1, panels A1 and B1). In the presence of Al^3+^, the absorption maximum of CIT shifted to λ_max_ = 365 nm (Figure 1, panel A1). The analysis of the photometric data with the Job plot method indicated a stoichiometry of the citrinin-aluminum complex (CIT-Al) of 2:1 (value of 0.66) (Figure 1, A2). A possible structure of the bidentate complex is given in Figure 2 (left). CIT possesses two 1,3-dicarbonyl moieties that are capable to chelate Al^3+^. This can lead to different complexes with the same metal-to-ligand ratios.

The complexation of OTA by Al^3+^ led to a shift of the absorption maximum from 332 nm to λ_max_ = 370 nm shown in Figure 1 (panel B1). The absorption spectra of OTA and Al^3+^ cross at an isosbestic point at 350 nm. The observation of isosbestic points indicates that only two optically active species are present in the solution, the free ligand, here the mycotoxin, and its complex, and that no side reaction occurs. The Job plot of the titration of OTA with Al^3+^ shown in Figure 1 (panel B2) revealed a value of 0.48 for the composition of the ochratoxin A-aluminum complex (OTA-Al) equaling a ratio of 1:1 for OTA and Al^3+^. OTA can bind to metal ions like Zn^2+^ and Mg^2+^ yielding 1:1 complexes with enhanced fluorescence [16]. In the OTA-magnesium complex, Mg^2+^ interacts with three partially negatively charged oxygen atoms of the mycotoxin: the phenolic oxygen, the oxo group oxygen, and the carboxylic group oxygen. Chelation of Zn^2+^ occurs via the nitrogen atom of the amide group and the two partially negatively charged oxygen atoms of OTA. This underlines that different moieties of OTA can be involved in metal ion coordination, depending on the specific requirements of the metal ions and potential steric constraints. In Figure 2, the chemical groups of OTA participating in Al^3+^ complexation are highlighted in red.

### 2.2. Fluorescence Characteristics

**Figure 3:**
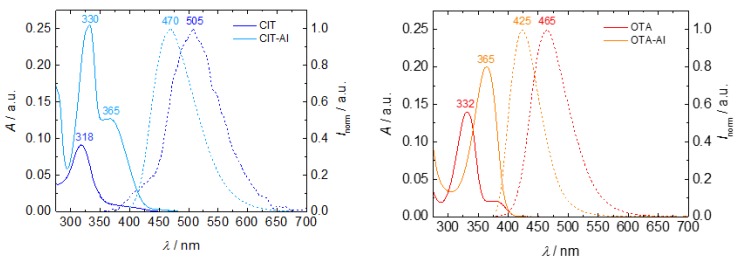
Absorption (solid lines) and normalized emission (dashed lines) spectra of citrinin (CIT) and ochratoxin A (OTA) and their Al^3+^ complexes in methanol (*c* = 30 µM).

**Figure 4:**
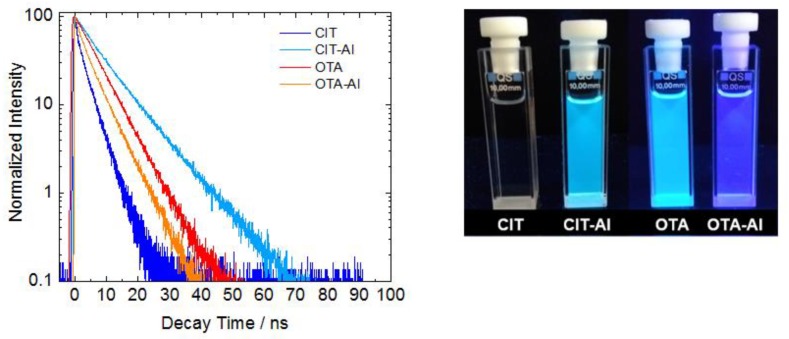
Left: fluorescence decay curves of citrinin (CIT), citrinin-Al-complex (CIT-Al), ochratoxin A (OTA), and ochratoxin A-Al complex (OTA-Al) providing the fluorescence lifetimes (τ); right: optical appearance of CIT, CIT-Al, OTA and OTA-Al (*c* = 50 µM in MeOH) under UV light.

The emission maxima of both complexes undergo hypsochromic shifts from 505 nm to 470 nm and 465 nm to 425 nm for CIT and OTA (Figure 3, dashed lines), respectively. Overall, the fluorescence of CIT is very weak (Figure 4, photo). The determination of the fluorescence quantum yield of CIT, which represents the ratio of the number of photons emitted to the number of photons absorbed and is a direct measure for fluorescence, yields a low value of 0.6% (Table 1). In contrast, CIT-Al showed a strongly increased fluorescence quantum yield of 29.5%. This chelation-induced increase in fluorescence can be detected by the naked eye, as shown in the photograph (Figure 4, right). This signal amplification can be used for a more sensitive detection of this mycotoxin using HPLC with fluorescence detection (FLD) as previously described for zearalenone and flavonoids [13,14]. For the OTA-Al complex, however, a decrease in fluorescence quantum yield from 44.7% to 34.2% was observed in the presence of Al^3+^ (Table 1), and the light blue fluorescence of OTA changed to a deep blue/violet fluorescence as shown in Figure 3 (right).

The enhancement in emission resulting for CIT is ascribed to the enlarged quinoide system generated by connecting two CIT molecules via Al^3+^. This also explains the much more pronounced red shift in the emission resulting for CIT-Al (Figure 3). The small influence of Al^3+^ coordination on OTA fluorescence is ascribed to the formation of a 1:1 complex, which barely affects the size of the π-electron system of this mycotoxin. The coordination-induced trends in fluorescence intensity are reflected by the results of the subsequently performed fluorescence decay measurements that yield the respective fluorescence lifetimes (τ) of the mycotoxin-Al^3+^ complexes. In the present study, we observed a fluorescence lifetime elongation for CIT-Al, whereas the complexation of OTA by Al^3+^ changed the ligand’s fluorescence lifetime only slightly (Figure 4, left and Table 1).

### 2.3. HPLC-FLD Measurements

**Figure 5:**
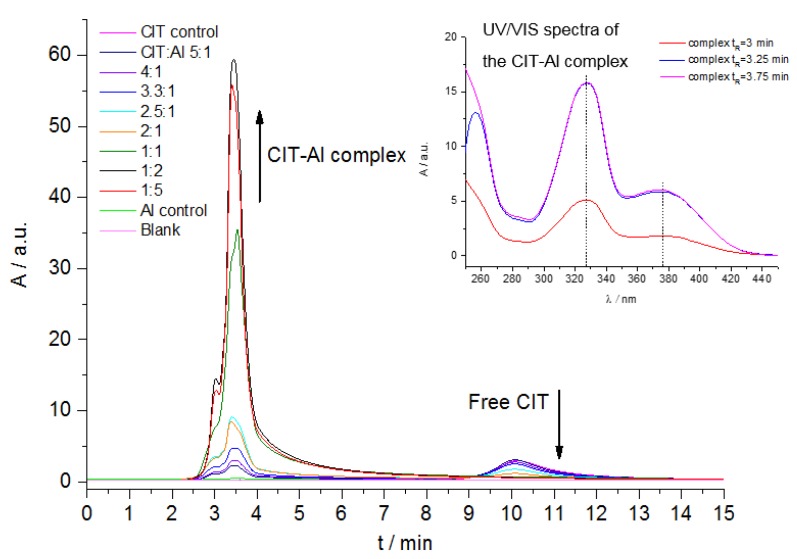
High performance liquid chromatography with fluorescence detection (HPLC-FLD) chromatograms of citrinin (*c*CIT = 50 µM) in the presence of varying molar ratios of Al^3+^ obtained with a PolymerX column; absorption spectra of citrinin-Al complex (CIT-Al) in acetonitrile/water obtained at different retention times measured with a diode array detector.

Chromatographic separation of metal complexes comprises several challenges. If the complexes are positively charged, they can adsorb onto the unreacted silanol groups of the reversed-phase column material and the elution is consequently inhibited or delayed. Furthermore, the disintegration of the complexes on a standard reversed-phase C18-HPLC column without endcapping reagents is possible. Consequently, pretests performed with C18-HPLC columns showed no clues for the separation of the Al^3+^ complexes from the respective mycotoxin ligands. However, the use of a polystyrene/divinylbenzene phase inhibits these effects and allows the separation of the metal complexes from the free ligands or metal ions [22]. Figure 5 shows the increase in fluorescence intensity resulting for the CIT-Al complex compared to free CIT, which equals an eightfold enhancement. Compared to the results of the fluorescence quantum yield measurements, the fluorescence enhancement is, however, lower than expected. The fluorescence of the CIT-Al complex seems to be reduced in the acetonitrile/water solvent mixture used for the HPLC studies compared to that in the methanol employed for the spectroscopic measurements. This was previously described for other aluminum complexes as well [19,20]. As the absorption spectra of the CIT-Al complex in acetonitrile/water and in methanol are rather similar (Figure 5, right), we assume that the same complex is formed in both solvents as major species. Nevertheless, we still also observe a considerable fluorescence enhancement in the HPLC-FLD studies, which is analytically very promising. As expected from the fluorescence spectroscopic studies, no signal enhancement was observed for OTA-Al.

## 3. Conclusions and Outlook

In summary, the coordination of the mycotoxins citrinin and ochratoxin A to trivalent Al^3+^ was studied photometrically and fluorometrically with special emphasis on chelation-induced fluorescence as tool for enhancing fluorescence signals and thereby decreasing the detection limit of HPLC-FLD analysis and quantification of CIT. Analysis of the photometric data of the titration of the mycotoxins with Al^3+^ in methanol with the Job method revealed the formation of a 1:1 complex of Al^3+^ and OTA and a 1:2 complex stoichiometry for Al^3+^ and CIT, with the participation of two CIT ligands causing a red shift in emission and a fluorescence enhancement of about 50. A less pronounced signal enhancement in the order of a factor of eight was obtained for high performance liquid chromatography with fluorescence detection (HPLC-FLD) using an acetonitrile/water solvent mixture. Thus, in the future, post-column derivatization of CIT-containing samples with Al^3+^ is expected to reduce the limit of detection for CIT analysis.

## 4. Materials and Methods

### 4.1. Chemicals

Citrinin (CIT) and ochratoxin A (OTA) with purities over 98% were obtained from Fermentek (Jerusalem, Israel). Al(NO_3_)_3_·9H_2_O was obtained from Merck (Darmstadt, Germany) and HPLC grade methanol and acetonitrile from Chemsolute (Th. Geyer, Renningen, Germany), respectively.

### 4.2. Absorption and Fluorescence Spectroscopy

Absorption spectra were recorded on a calibrated Varian Cary 5000 UV-VIS-NIR spectrometer (Agilent Technologies GmbH, Waldbronn, Germany) with a scan rate of 300 nm/min and a slit width of 1 nm using a baseline correction (air/air) and a solvent sample (methanol) as reference. Fluorescence data were collected on a calibrated FluoroMax-4P fluorometer from HORIBA Jobin Yvon (Edison, NJ, USA) with an integration time of 0.1 s and slit widths of 2 nm for excitation and emission.

### 4.3. Fluorescence Quantum Yields (Φ)

*Φ* values, which represent the ratio of the number of emitted photons per number of absorbed photons, were determined absolutely with an integrating sphere setup from Hamamatsu (Quantaurus-QY C11347-11, Hamamatsu, Japan) as described previously [23]. All *Φ* measurements were performed at 25 °C using special 10 mm × 10 mm long neck quartz cuvettes from Hamamatsu.

### 4.4. Time-Correlated Single Photon Counting (TCSPC)

Fluorescence decay kinetics providing the fluorescence lifetimes (*τ*) of the mycotoxins and mycotoxin-aluminum complexes were recorded with a FLS 920 fluorometer from Edinburgh Instruments (Edinburgh, UK) equipped with a 330 nm or 375 nm pulsed light-emitting diode of the EPLED series and a fast-multichannel plate photomultiplier (MCP-PMT) as a detector. The samples of CIT and OTA were excited at 330 nm and the samples of CIT-AL and OTA-Al were excited at 375 nm, while the emission was detected at the respective emission maximum employing a spectral bandwidth of the excitation and emission monochromator (Edinburgh Instruments, Edinburgh, UK) of 15 nm, a 4096-channel setting, and time ranges of 100 ns and 200 ns, respectively. With this setup, *τ* values ≥0.2 ns can be reliably measured. The measured fluorescence decay kinetics were evaluated using the deconvolution procedure of the FAST program. This procedure considers the measured instrument response function (IRF), which influences the fluorescence decays. All decay profiles could be analyzed with mono-exponential fits with reduced *χ*^2^ values between 0.8 and 1.2.

All spectroscopic measurements were performed with air saturated solutions at *T* = 25 °C using 10 mm × 10 mm quartz cuvettes from Hellma GmbH (Muellheim, Germany) filled with 3 mL of solvent or mycotoxin solution to be analyzed.

### 4.5. Job Plot Analysis

For the application of the Job plot analysis, citrinin, ochratoxin A, and Al(NO_3_)_3_ were dissolved in methanol, obtaining stock solutions with *c* = 100 µM, respectively. Different mole fractions of ligand and metal were prepared in which the sum of the total concentration of mycotoxin and Al^3+^ remained constant (100 µM), but their proportions were continuously varied. For example, the mole fraction (ligand:metal) of 0.4 was prepared with 800 µL of mycotoxin stock solution and 1200 µL of Al(NO_3_)_3_ solution. The resulting Job plot working solutions of 2 mL were measured photometrically after 10 min of incubation at room temperature. All experiments were performed at least three times.

### 4.6. HPLC-DAD/FLD Measurements

The mycotoxin-aluminum complexes were analyzed by HPLC using an Agilent 1200 series HPLC (Agilent Technologies GmbH, Waldbronn, Germany) consisting of an auto sampler, a binary pump, a degasser, a column oven, a diode array (DAD), and fluorescence detector (FLD). The analytical column used was a PolymerX RP-1 with dimensions of 250 × 4.6 mm, a particle size of 5 µm and a pore size of 100 Å (Phenomenex, Torrance, CA, USA) and the column oven was set to 40 °C. The mobile phase consisted of 60% acetonitrile and 40% water without modifiers for the CIT-Al HPLC method, and for the OTA-Al method, 100% of methanol were used. For both methods, the flow rate of the mobile phase was 800 µL/min, the injection volume was 20 µL, and the isocratic run was held for 15 min. A DAD scan was performed in the wavelength range of *λ* = 190–800 nm, and the FLD was set to excitation and emission wavelengths as follows: CIT *λ*_Ex_ = 331 nm and *λ*_Em_ = 500 nm; CIT-Al complex *λ*_Ex_ = 320 nm and *λ*_Em_ = 474 nm; OTA *λ*_Ex_ = 330 nm and *λ*_Em_ = 465 nm; OTA-Al complex *λ*_Ex_ = 365 nm and *λ*_Em_ = 425 nm.

## Figures and Tables

**Table 1:** Spectroscopic properties of the mycotoxins citrinin (CIT), ochratoxin A (OTA) and their Al^3+^ complexes in methanol.

Analyte	*ε*(λ_max_)/L·mol^−1^·cm^−1^	*λ*_em_/nm	*Φ*/%	*τ*/ns
CIT	3030 (318)	505	0.6	3.7
CIT-Al	8440 (330); 4220 (365)	470	29.5	9.7
OTA	4650 (332)	465	44.7	6.3
OTA-Al	6700 (365)	425	34.2	5.5

*ε*(λ_max_) = molar extinction coefficient, *λ*_em_ = wavelength of emission maximum, *Φ* = quantum yield, *τ* = fluorescence lifetime.
